# Two paralogous EcfG σ factors hierarchically orchestrate the activation of the General Stress Response in *Sphingopyxis granuli* TFA

**DOI:** 10.1038/s41598-020-62101-z

**Published:** 2020-03-20

**Authors:** Rubén de Dios, Elena Rivas-Marin, Eduardo Santero, Francisca Reyes-Ramírez

**Affiliations:** Centro Andaluz de Biología del Desarrollo, Universidad Pablo de Olavide/Consejo Superior de Investigaciones Científicas/Junta de Andalucía. Departamento de Biología Molecular e Ingeniería Bioquímica, Seville, Spain

**Keywords:** Bacterial physiology, Bacterial transcription

## Abstract

Under ever-changing environmental conditions, the General Stress Response (GSR) represents a lifesaver for bacteria in order to withstand hostile situations. In *α-proteobacteria*, the EcfG-type extracytoplasmic function (ECF) σ factors are the key activators of this response at the transcriptional level. In this work, we address the hierarchical function of the ECF σ factor paralogs EcfG1 and EcfG2 in triggering the GSR in *Sphingopyxis granuli* TFA and describe the role of EcfG2 as global switch of this response. In addition, we define a GSR regulon for TFA and use *in vitro* transcription analysis to study the relative contribution of each EcfG paralog to the expression of selected genes. We show that the features of each promoter ultimately dictate this contribution, though EcfG2 always produced more transcripts than EcfG1 regardless of the promoter. These first steps in the characterisation of the GSR in TFA suggest a tight regulation to orchestrate an adequate protective response in order to survive in conditions otherwise lethal.

## Introduction

In an environment where conditions are constantly changing, bacteria need to adapt their physiology in order to increase their chances of survival. These adaptations are not only mediated by particular responses to certain stimuli, but also by global changes in the transcriptional profile. These changes allow bacteria to thrive in conditions that otherwise would be considered hostile habitats for them. Global transcriptional adaptations can be exerted by a number of mechanisms, all of them ending up in a regulator that allows or prevents the expression of certain genes^1^. One direct way to alter the transcription is the swapping of σ factors, small switchable subunits of the RNA polymerase (RNAP) whose main functions are the promoter recognition and the double strand unwinding to form the transcription bubble^2^. The general classification of σ factors comprises two main groups: the σ^70^ and the σ^54^ families. Among all of them, the vegetative σ^70^ factor, responsible for transcribing the bulk of bacterial genes, is the only essential representative^3^. The σ^70^ family can be subdivided into four groups based on their domain architecture. This include the σ^70^ housekeeping Group I, and the non-essential structurally-related alternative σ factor Groups II–IV, which despite their large variation in size, all keep the σ_2_ and σ_4_ domains, responsible for binding the -10 and -35 boxes, respectively, in a promoter region^2,3^. Extracytoplasmic function σ factors (ECFs), belonging to Group IV, are the minimal expression of this family, since they are only formed by the σ_2_ and σ_4_ domains, but are also the most abundant and diverse in the bacterial world^1^. From a physiological point of view, they participate in regulating the transcription in response to a wide range of environmental signals^4^.

In *α-proteobacteria*, ECFs play a central role in the regulation of the so-called General Stress Response (GSR). This protective response is activated by a number of non-specific stresses, such as desiccation, osmotic shock, oxidative stress or carbon starvation among others^5^. Three main regulatory elements are required to control the GSR in *α-proteobacteria*: an EcfG-type ECF σ factor (G standing for GSR), its cognate anti-σ factor NepR and the response regulator PhyR^4^. In favourable growth conditions, EcfG is sequestered by NepR, thus preventing the expression of the GSR regulon. When some kind of stress appears in the environment, one or more GSR-specific histidine kinases belonging to the HWE or HisKA2 family start the signal transduction by autophosphorylation in a His residue and transfer the phosphoryl group to an Asp residue in PhyR^6^. This phosphorelay causes a conformational change in PhyR, so that a σ-like domain is exposed. Upon this, by a partner-switching mechanism, NepR is sequestered by the σ-like domain presented by PhyR, thus releasing EcfG to activate the expression of the GSR target genes^6^. This model may display a diverse variety of organizations, with different levels of complexity according to the number of PhyR, NepR and EcfG paralogs encoded in the genome^7–10^ and the presence of accessory regulators with different levels of essentiality for the activation of the GSR^11–13^.

Regarding EcfG, most of the described models only have one or two paralogs^14,15^, although more baroque models, such as the one for *Methylobacterium extorquens*, have been described^10^. With respect to the function and the distribution of the target genes among the different EcfG copies, there are diverse examples described, such us in *Rhizobium etli*, where the two EcfG proteins show some sort of functional specialisation between them by controlling different sets of genes^14^, or *Caulobacter crescentus*, in which the additional EcfG copy σ^U^ controls to some extent the expression of a small subset of genes belonging to the regulon of the main regulator σ^T^, putatively to amplify its response^15,16^. In other models, the number of EcfG elements can be even higher, such as the case of *M. extorquens*, where up to six of these regulators were annotated. Nevertheless, a main regulator could be identified as EcfG1^10^.

The α-proteobacterium *Sphingopyxis granuli* TFA is one of the few strains able to use the organic solvent tetralin as a carbon and energy source. Tetralin catabolism has been intensively characterised in this bacterium at biochemical, genetic and regulatory level^17^. Moreover, TFA is the only strain within its genus that has been reported to anaerobically grow while respiring nitrate^18,19^. Recently, its genome has been totally assembled and annotated, showing some characteristics of oligotrophic bacteria^18^.

In this work, we have identified in the TFA genome two paralogous genes encoding EcfG σ factors and defined their hierarchical role as regulators of the GSR. We have also identified the members of the GSR regulon in TFA using transcriptomic analyses.

## Results and Discussion

### *S. granuli* TFA presents two GSR regulatory σ factors

**Figure 1:**
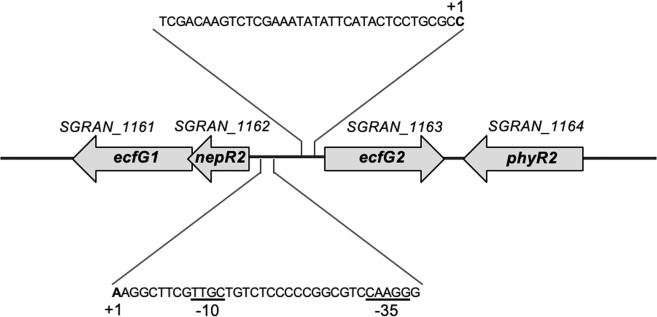
Genetic organization of the *nepR2ecfG1*-*ecfG2* divergent *orfs*. Putative promoter regions according to previously reported transcription start sites (TSS) are highlighted above the genes for *ecfG2* and below for the operon *nepR2ecfG1*. TSS (+1) are marked in bold, and putative -10 and -35 boxes for GSR consensus target promoters appear underlined. Arrowheads indicate the direction of transcription.

We have analysed the recently annotated TFA genome searching for candidates with a common signature of ECF σ factors: the characteristic architecture with only domains σ_2_ and σ_4_, connected by a short unstructured linker (<50 amino acids). TFA bears a total of 25 genes encoding putative ECF σ factors that might play a regulatory role in its physiology. Using the classification tool developed by Staron *et al*.^1^, which allows the analysis of the ECF by sequence similarity for further assignment to distinct functional categories, two of them were classified in the ECF15 category. This unique group is formed exclusively by EcfG-type σ factors whose main function is the activation of the GSR in *α-proteobacteria*, given that this taxonomic group lacks *rpoS* homologs^5,20^. Therefore, the corresponding genes *SGRAN_1161* and *SGRAN_1163* were subsequently designated as *ecfG1* and *ecfG2*, respectively. One of the main features of the EcfG group is a conserved genomic context consisting in the EcfG-like σ factor preceded by an anti-σ factor (NepR-like proteins) and with a PhyR-like response regulator in the vicinity, which functions as the anti-anti-σ factor^5^. Although *nepR* genes are not usually automatically annotated due to their small size, the TFA genome revealed that it contains two short *orfs* that could actually code for NepR-like proteins and were therefore designated as *nepR1* (*SGRAN_0992*) and *nepR2* (*SGRAN_1162*). The one designated *nepR2* is located upstream *ecfG1*, shares the same transcription start site (TSS)^21^ and actually overlaps with *ecfG1* by 4 bp (Fig. 1). A putative EcfG target promoter (GGAAC-N_16_-CGTT) was found upstream *nepR2*, suggesting that these genes form an operon that could be auto-regulated, an organization previously reported for other GSR regulatory σ factors in *α-proteobacteria*^6^. Divergently from this transcriptional unit, the *ecfG2* gene is located, which does not bear the proposed consensus for EcfG-type promoters and does not present a cognate anti-σ factors upstream (Fig. 1). Next to *ecfG2*, in a convergent orientation, one of the *phyR* (*phyR2, SGRAN_1164*) elements present in TFA is found, completing the typical synteny conserved in *α-proteobacteria*, in which at least one representative of each GSR regulatory element is encoded in the same locus^6^. On the other hand, the second putative *nepR* paralog, *nepR1*, is located elsewhere in the genome divergently from a second *phyR* element (*phyR1*, *SGRAN_0993*).

The presence of paralogous regulatory elements in the α-proteobacterial GSR cascades has emerged as an usual feature based on the analysis of multiple of α-proteobacterial genomes^5^. However, the relationships among the different regulators when multiple copies of them are present seem to be species-specific. To start the characterisation of the GSR cascade in TFA, we first focused on the ECF σ factors EcfG1 and EcfG2.

### EcfG2 is the main GSR regulator in *S. granuli* TFA

**Figure 2:**
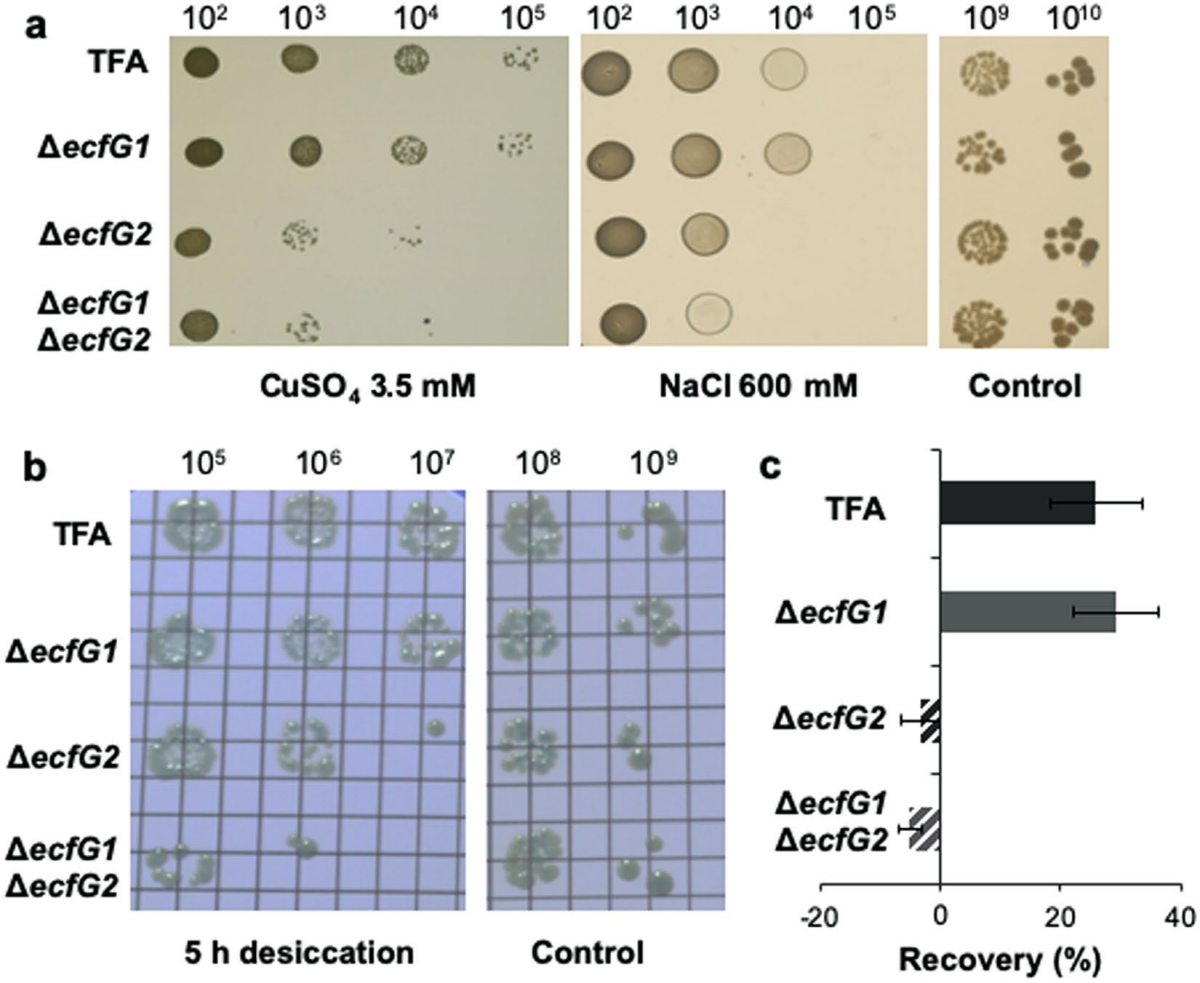
Phenotypic stress assays comparing the different *ecfG* mutants with the wild type TFA. Stress sensitivity was measured spotting serial dilutions of the different strains on agar MML rich medium supplemented with CuSO_4_ 3.5 mM or NaCl 600 mM (**a**). Sensitivity to desiccation was measured by spotting serial dilutions on filters that were left to air dry for 5 h (**b**). The capability to recover from oxidative shock was measured comparing cultures with the untreated controls 5 h after the addition of H_2_O_2_ 10 mM (**c**).

As mentioned above, the GSR in *α-proteobacteria* is mediated by EcfG homologs, which belong to the ECF15 class. In order to evaluate whether EcfG1 and EcfG2 play a protective role in response to environmental stresses in TFA and whether they are redundant in function, Δ*ecfG1*, Δ*ecfG2* and double Δ*ecfG1*Δ*ecfG2* deletion mutants were constructed and their resistance to different stresses, such us, heavy metals, osmotic stress, desiccation and exposure to hydrogen peroxide, was tested. No growth differences were observed between the wild type and the Δ*ecfG1* mutant on agar plates in the presence of copper 3.5 mM, NaCl 600 mM (Fig. 2A), or after 5 h desiccation (Fig. 2B), and they show the same capability to recover from oxidative shock after adding H_2_O_2_ in mid-exponential phase (Fig. 2C). However, a decreased viability was observed for the Δ*ecfG2* mutant after copper, salt, desiccation and H_2_O_2_ stresses, showing higher sensitivity to all these treatments than the wild type and the strain lacking EcfG1 (Fig. 2). The double Δ*ecfG1*Δ*ecfG2* mutant strain displayed similar level of sensitivity to all the treatments than the Δ*ecfG2* mutant strain (Fig. 2), although its sensitivity to NaCl and desiccation might be slightly higher (Fig. 2A,B). These results clearly show that the absence of EcfG1 does not impair the resistance of TFA to the stresses tested, being EcfG2 the main responsible for the stress resistance.

In previous works, when several *ecfG* copies are present^7,10,16^, some sort of interdependency among them has been shown, so TFA may present a similar situation. For this reason, we next focused on how the expression of *ecfG1* and *ecfG2* is regulated and whether there could be some sort of interdependency between them.

### EcfG2 hierarchically controls the expression of *ecfG1*

**Figure 3:**
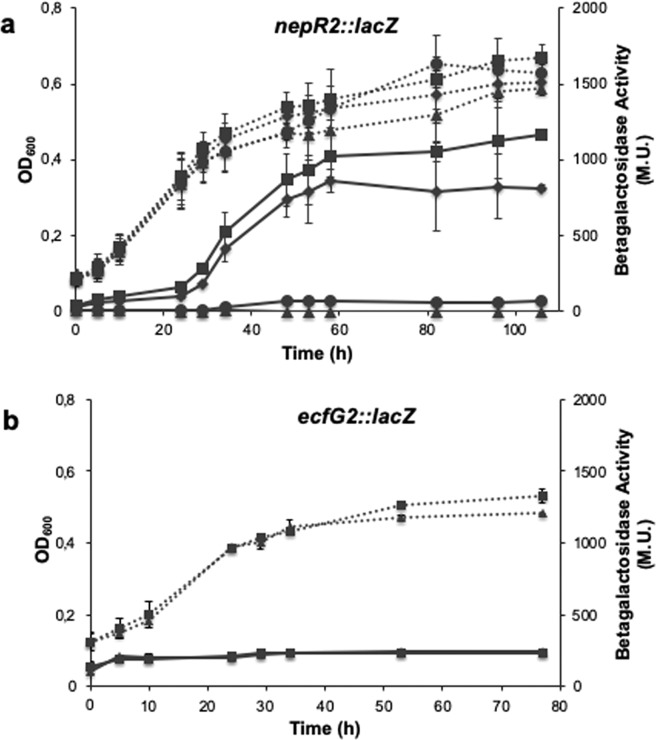
Expression patterns of *ecfG1* and *ecfG2* in the WT and mutant strains throughout the growth curve. β-galactosidase activity (whole lines) of a *nepR2::lacZ* fusion during growth of WT TFA (squares), Δ*ecfG1* (rhombi), Δ*ecfG2* (circles) and Δ*ecfG1*Δ*ecfG2* (triangles) mutant strains (**a**). β-galactosidase activity (whole lines) of a *ecfG2::lacZ* fusion in the WT (squares) and Δ*ecfG1*Δ*ecfG2* (triangles) strains (**b**). Dotted lines represent OD_600_ of the different strains over time.

As it has been described for other members of the *α-proteobacteria*, carbon starvation is an inducing condition of the GSR^22^. Therefore, to study the expression of *ecfG1* and *ecfG2* in response to stress, we started by monitoring their expression along a growth curve in minimal medium with 8 mM of BHB as a carbon source instead of 40 mM, which is used for normal growth^23^, so that the culture enters stationary phase as a consequence of carbon exhaustion. In order to do this while having an indication of the level of activation of the GSR, two translational fusions were constructed by cloning DNA fragments containing the putative promoters, the ribosome binding site and the first codons of either *nepR2* (the first gene of the *nepR2ecfG1* operon) or *ecfG2* upstream the *lacZ* gene in an integrative vector. After the introduction of the fusions in TFA, as shown in Fig. 3A, a basal expression of *ecfG1* was observed during the exponential phase of growth, which was induced over 6-fold when stationary phase was reached. However, the expression of *ecfG2* was constitutive regardless of the growth stage (Fig. 3B).

Since one of the common features of the EcfG σ factors is their transcriptional autoregulation^4^, both reporter fusions were also introduced in the different *ecfG* single and double mutant strains presented above, and the expression was measured throughout the growth curve. None of these mutant strains showed differences in growth when compared to the wild type (Fig. 3). Data shown in Fig. 3A indicate that *ecfG1* expression was only moderately affected in an *ecfG1* mutant, maintaining around 80% of the activity, but it was dramatically affected in an *ecfG2* mutant, with less than 5% of the wild type activity. No detectable expression was obtained in the double Δ*ecfG1*Δ*ecfG2*. These results indicate that *ecfG1* expression increases by entry into stationary phase by carbon starvation in an EcfG2-dependent manner, and that EcfG1 could have a mild positive auto-regulation. These results were expected, considering its −35 and −10 promoter motives, characteristic of the EcfG-dependent promoters. In contrast to *ecfG1*, as shown in Fig. 3B, *ecfG2* expression remained invariant during growth even in an Δ*ecfG1*Δ*ecfG2* double mutant background. These data indicate that, although stationary phase is an EcfG2 activating condition, *ecfG2* expression is not stress-responsive, and that neither EcfG1 nor EcfG2 itself are essential for the expression of *ecfG2*, in agreement with the absence of a putative EcfG-type sequence in the *ecfG2* promoter region.

Altogether, these expression data, coherently with the resistance phenotypes shown above (Fig. 2), would suggest a model in which EcfG2 would be the global switch of the response, whose expression would be constitutive, and with *ecfG1* depending entirely on the EcfG2-dependent regulation to be expressed. This resembles the model described for *C. crescentus*^16,22^, in which σ^T^ regulates the expression of σ^U^, with the difference that σ^T^ is also autoregulated, in contrast to EcfG2 in TFA.

### The GSR regulon comprises 79 transcriptional units in *S. granuli* TFA

Even though the GSR-activated genes have been elucidated for different species, the global physiological implications of this response as a whole have remained a matter of discussion, given the poor functional annotation of many genomes in the *α-proteobacteria* phylum, and more specifically in the *Sphingomonadaceae* family.

In this regard, and in order to characterise the global transcriptional behaviour mediated by the GSR in TFA, transcriptomic analyses were performed by dRNA-seq comparing an Δ*ecfG1*Δ*ecfG2* double mutant, unable to activate the GSR, to a wild type strain under carbon starvation conditions, a situation in which the GSR is triggered as described in the previous section. As a result, after applying a cutoff of ≥3-fold change in expression, a total of 189 coding genes were downregulated and 257 coding genes were upregulated (4.5% and 6.1%, respectively, of all coding genes annotated in the *S. granuli* TFA genome, and almost 50% of them with unknown function) in the Δ*ecfG1*Δ*ecfG2* mutant compared to the wild type (see Supp. Table S1 and Supp. Fig. S1). The putative function of the differentially expressed genes was inferred using the current annotation of the TFA genome and the Cluster of Orthologous Groups of proteins broad classification (COG)^24^.

**Figure 4:**
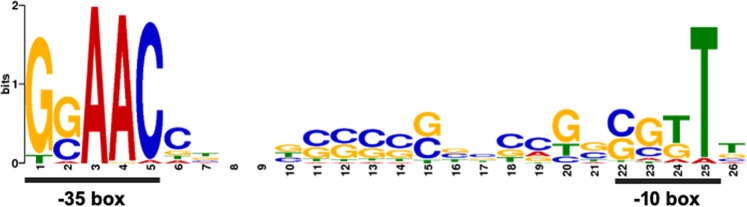
GSR target motif obtained for *S. granuli* TFA. The consensus sequence logo was generated with the tool MEME^25^ using as input the putative promoter sequences of genes found downregulated in the dRNA-seq analysis comparing the Δ*ecfG1*Δ*ecfG2* mutant to the wild type strain. Whereas the -35 box is highly conserved, the -10 box shows certain flexibility.

Given that some of the genes found downregulated in the transcriptomic study might be affected in a GSR-independent manner, a promoter sequence analysis was performed in order to define a GSR direct regulon. To do this, the DNA regions comprising the 450 bp upstream the 189 genes downregulated in the Δ*ecfG1*Δ*ecfG2* mutant were analysed using the motif discovery tool MEME^25^, as described in Materials and Methods. Expectedly, the conventional GSR promoter motif GGAAC-N_16_-CGTT, as shown in Fig. 4, was found enriched among the promoter sequences. After that, all the sequences were manually inspected using as a guide the annotation and the previously identified TSSs^21^, and those genes with an unlikely promoter organisation or which did not contribute clearly to the detected motif were discarded. As a result, a GSR regulon with 104 coding genes (55% of all the downregulated genes) distributed in 79 putative operons was defined for *S. granuli* TFA (see Supp. Table S2 and Supp. Fig. S2).

Several GSR regulons have been defined in diverse α-proteobacterial species, including *C. crescentus*^15^, *Sinorhizobium. meliloti*^9^*, M. extorquens*^26^, *Bradyrhizobium japonicum*^27^, *R. etli*^28^ and *Sphingomonas melonis*^13^, and a common feature in all of them is the high abundance of target genes with unknown function, a situation also described here for TFA (only 37% of the GSR-dependent genes have a predicted function). Among the genes directly regulated by the GSR, some obvious targets could be found, such as most of the regulatory elements of the response (the *nepR2ecfG1* operon, *phyR1* and *phyR2*, and the HWE family histidine kinase *SGRAN_1165* located next to *phyR2*), as described in the literature^4^. Noticeable examples of genes directly regulated by the GSR among the genes with annotated function are the stress inducible *csbD* (*SGRAN_3063*), two *katA* catalases (*SGRAN_1770* and *SGRAN_2521*)^4^, the *ecnAB* toxin-antitoxin system^29^ (*SGRAN_1922*), an *mscS* mechanosensitive ion channel^30^ (*SGRAN_0895*), an *osmC* peroxiredoxin^31^ (*SGRAN_3924*), an RND efflux system component^15^ (*SGRAN_4134*) and the *ku-ligD* non-homologous DNA repair system (*SGRAN_4136* and *SGRAN_4135*)^32^, some of them recently found GSR-dependent in the sphingomonad *S. melonis*^13^. In addition, a large representation of envelope related elements also appear in this regulon (e.g., *SGRAN_1221*, *SGRAN_1797*, *SGRAN_1798*, *SGRAN_2253*, *SGRAN_2364*, *SGRAN_2492* or *SGRAN_2662*)^4^. However, some other elements, such as the sphingosine kinase coding gene (*SGRAN_0919*)^33^ or the putative *egtB* and *egtD* genes (*SGRAN_4050* and *SGRAN_4049*), which may be involved in ergothioneine biosynthesis^34^ are reported as part of a GSR regulon for the first time.

Together with the genes directly regulated by the GSR, a number of genes altered their expression independently of this response, maybe as an indirect regulation mediated by the GSR or perhaps as an adaptation to a hostile situation that could not be properly overcome because of the double deletion. As shown in Supp. Fig. S1, the expression of some metabolic genes, signalling elements and putative transcriptional regulators was altered in the absence of GSR. Another remarkable global change was the upregulation of the flagellar clusters and homologous genes to flagellar regulators, such as a putative *fleQ* (*SGRAN_4107*) and *ctrA* (*SGRAN_3324*), and some elements involved in chemotaxis, such as *cheA* (*SGRAN_2956*)*, cheB* (*SGRAN_2953*) or *cheR* (*SGRAN_2952*)^35^, together with a putative *bdlA* biofilm dispersal regulator^36^. This phenomenon may imply a mechanism to escape from unfavourable situations, such as carbon starvation, because of the absence of a functional stress response.

Altogether, our transcriptional analysis shows that many cellular processes may be affected to cope with stress, having the GSR a key role in the protection. Also, given that all the genes manually inspected, *a priori* presented as direct GSR targets, revealed the same promoter consensus sequence, this may suggest a model in which both ECFs regulate the same genes but with one of them having a secondary role. To address this, we next focused on the capability of EcfG1 and EcfG2 to recognise selected promoters and their differences in the regulation.

### EcfG1 might contribute differently to the transcription depending on the target

**Figure 5:**
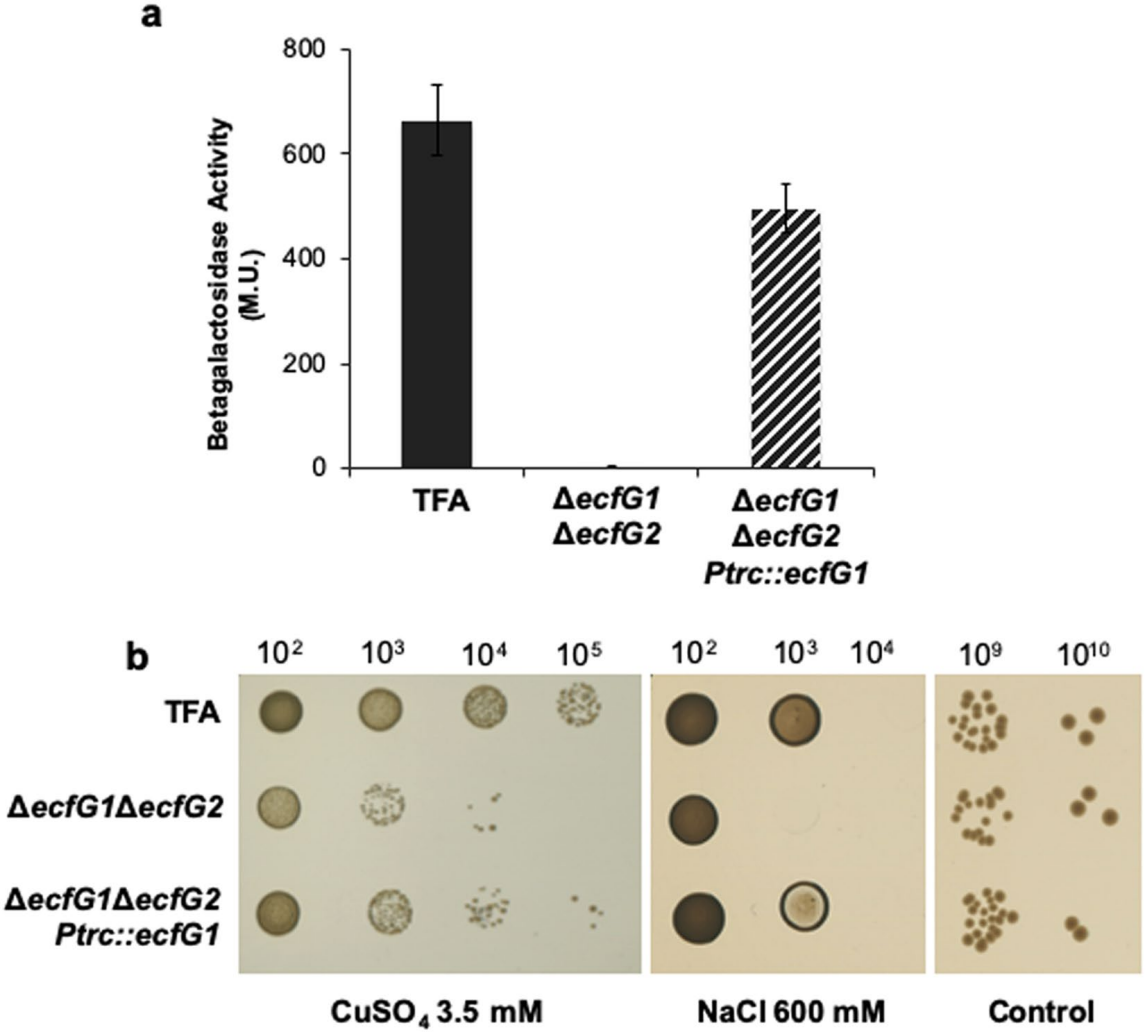
Ectopic expression of *ecfG1* in an *ΔecfG1ΔecfG2* mutant background compared to the wild type strain. A partial recovery of the expression from the *P*_*nepR2*_ promoter was shown upon entrance in stationary phase (**a**). Also, a partial phenotypic complementation was observed regarding sensitivity to CuSO4 3.5 mM and NaCl 600 mM (**b**). Control strains harboured plasmid pSEVA224, while the complemented strain carried pMPO1433, in all the cases in the presence of IPTG 1 mM.

As *ecfG1* expression is dependent on EcfG2, it may be considered that the absence of stress protection in an *ecfG2* mutant (Fig. 2) might be caused by low levels of *ecfG1* expression in that background. Thus, the effects of ectopic *ecfG1* expression on transcriptional activation of the *ecfG1* own promoter and on its tolerance to heavy metals and osmotic stresses was examined (Fig. 5). To do that, plasmid pMPO1433, with the *ecfG1* coding sequence under the IPTG inducible (thus, EcfG2-independent) *P*_*trc*_ promoter, was constructed and introduced into the Δ*ecfG1*Δ*ecfG2* double mutant containing the *nepR2::lacZ* translational fusion inserted in the chromosome. As control, a wild type strain and the Δ*ecfG1*Δ*ecfG2* mutant carrying the parental plasmid were also included in the analysis. Activity from *ecfG1* promoter in the complemented strain was determined in cultures grown to stationary phase in the presence of IPTG. Figure 5A shows that, when EcfG1 was produced independently from EcfG2, the expression of *ecfG1* own promoter was induced to similar levels to the wild type strain. These results confirm that EcfG1 is able to substitute EcfG2 in activating its own expression if ectopically produced in sufficient amounts in the cell.

Coherently with the results mentioned above, Fig. 5B shows that ectopic expression of *ecfG1* resulted in an increased resistance to heavy metal and salt stresses when compared to the Δ*ecfG1*Δ*ecfG2* double mutant. However, the complemented strain did not reach the levels of tolerance observed in the wild type strain, indicating only a partial stress resistance. These results indicate that EcfG1 has the capability to partially activate the GSR in TFA on its own when produced in sufficient amounts, thus suggesting that it may play a secondary role in this stress resistance pathway.

The partial resistance obtained in the sole presence of EcfG1 might indicate regulatory differences between EcfG1 and EcfG2 in a promoter-specific manner. In order to address this, the transcript levels of a number of genes were quantified by RT-qPCR in the single and double *ecfG* mutant backgrounds, and also in the Δ*ecfG1*Δ*ecfG2* mutant with *ecfG1* ectopically expressed, and compared to the respective wild type strain in stationary phase. For that, ten genes, identified as members of the GSR regulon, were selected, including *nepR2*, whose expression pattern was already described by *lacZ* translational fusions. As control, we tested the expression of *ecfG2*, which had already been demonstrated to be constitutive and GSR-independent (Fig. 3). As shown in Table 1, *ecfG2* expression remained practically unaffected in all the genetic backgrounds. EcfG2 was the main contributor in all promoters. In contrast, EcfG1 showed different levels of contribution at different promoters, as shown by either comparing the Δ*ecfG1* mutant to the wild type strain or the Δ*ecfG1*Δ*ecfG2* double mutant ectopically expressing *ecfG1* to the wild type bearing the parental plasmid. Considering that the effective levels of EcfG1 are the same regardless of the gene tested, these results might indicate that the relative contribution of each σ factor would depend on the characteristics of each promoter, as well as on the transcription potential of each EcfG paralog.

**Table 1:** Expression quantification of selected genes in different *ecfG* mutant backgrounds.

Gene	Δ*ecfG1*^a^	Δ*ecfG2*^a^	Δ*ecfG1*Δ*ecfG2*^a^	Δ*ecfG1*Δ*ecfG2*^b^ *P*_*trc*_*::ecfG1*
*csbD (SGRAN_3063)*	0.52 ± 0.03	0.01 ± 0.00	0.00	1.88 ± 0.06
*ecnAB (SGRAN_1922)*	0.68 ± 0.05	0.03 ± 0.00	0.00	1.36 ± 0.11
*gsp (SGRAN_0888)*	0.79 ± 0.03	0.01 ± 0.00	0.03 ± 0.00	2.39 ± 0.46
*ku (SGRAN_4136)*	1.34 ± 0.07	0.16 ± 0.01	0.16 ± 0.03	0.57 ± 0.02
*mltA2 (SGRAN_1260)*	1.3 ± 0.11	0.00	0.00	0.26 ± 0.01
*nepR2 (SGRAN_1162)*	0.44 ± 0.04	0.02 ± 0.00	0.00	0.74 ± 0.06
*SGRAN_1766*	1.10 ± 0.02	0.05 ± 0.00	0.05 ± 0.00	0.22 ± 0.00
*SGRAN_2195*	1.15 ± 0.14	0.06 ± 0.00	0.03 ± 0.00	1.78 ± 0.01
*SGRAN_2273*	0.91 ± 0.01	0.02 ± 0.00	0.01 ± 0.00	2.64 ± 0.10
*yiaD (SGRAN_3949)*	1.26 ± 0.13	0.04 ± 0.00	0.04 ± 0.00	0.19 ± 0.03
*ecfG2 (SGRAN_1163)*	0.86 ± 0.10	0.78 ± 0.22	0.86 ± 0.16	1.17 ± 0.13

Values represent fold-changes in expression with respect to the wild type strain in the case of the deletion mutants (a) or a wild type bearing plasmid pSEVA224 in the presence of IPTG 1 mM in the case of the double mutant ectopically expressing *ecfG1* (b). As control, the expression of *ecfG2*, which is GSR-independent, was also measured. Errors are calculated as the standard deviation of three independent replicates.

Even though the deletion of *ecfG1* does not seem to cause an observable impact on the resistance phenotype (Fig. 2), these expression data indicate that, for some targets, the transcription driven by EcfG1 represents a substantial fraction of the total expression, which may indicate that EcfG1 is needed to trigger a robust GSR. This is of capital importance in changing environments, in which bacteria need to quickly adapt to new situations in order to survive. Nevertheless, in the conditions tested, the GSR triggered by EcfG2 is enough to cope with stress, whereas EcfG1 alone can protect only partially, most likely as an effect of its modest contribution to the expression of a certain subset of genes.

### The GSR-dependent promoters show flexibility in the -10 box

Given the results mentioned above, which could indicate different relative contributions of EcfG1 to the transcription depending on the target gene, a detailed analysis of the GSR-dependent putative promoter sequences was performed. In all the studied promoters, a clear tendency to have a conserved -35 box with sequence GGAAC was observed, as shown in Fig. 4 (see also Supp. Table S2). As previously reported for ECF sigma factors^3^, the AAC triplet appeared almost invariant, since only 3 out of 79 of the putative -35 boxes presented one mismatch in that trinucleotide. On the other hand, much more flexibility was observed in the -10 box (as it can be appreciated in Fig. 4), since up to 14 different putative -10 boxes were identified among the GSR regulon, with CGTT being the most frequent. This might imply different scenarios involving the capability of being activated by EcfG1 and/or EcfG2.

As mentioned above, many species encode multiple EcfG paralogs and much effort has been made trying to establish possible regulatory differences among them, either by transcriptomic studies or at the level of gene expression phenotype. Since these approaches did not always result in obvious differences, we have introduced *in vitro* transcription (IVT) assays in order to distinguish between EcfG1 and EcfG2 at the molecular level.

### EcfG2 activates transcription at higher levels than EcfG1, regardless of the promoter

All the results shown above indicate that EcfG2 has a major role in the GSR in TFA both at the level of gene regulation and in resistance to different stresses, thus relegating EcfG1 to a secondary role as an accessory activator. In order to know the specific contribution of EcfG1 and EcfG2 to the transcription of different GSR promoters, *in vitro* transcription assays were performed with either EcfG1 or EcfG2 forms of the RNAP using five putative promoter regions, representatives of the most frequent -10 boxes, as templates. In order to restrict the regulatory inputs to the effect of the σ factor alone, the DNA regions used as templates would only extend from the putative TSS^21^ to the -35 sequence. Among the chosen templates, *nepR2* and *gsp* (general stress protein*, SGRAN_0888*) were selected for having a consensus CGTT -10 box, but also for differing in the -35 element (they present a GGAAC and a GCAAC -35 box, respectively, the two most frequent in the GSR-dependent promoters, see Supp. Table S2). Therefore, this would rule out the possibility that the -35 sequence might also be involved in the expression phenotype described in previous sections. The rest of the selected promoters were representatives of the most frequent -10 boxes apart from the consensus CGTT: the envelope related gene *yiaD* (*SGRAN_3949*), the putative transglycosylase *mltA2* (*SGRAN_1260*) and *SGRAN_2273*, which putatively contains an EF-hand Ca-binding domain, harbouring the -10 boxes TGTT, CATT and GGTT, respectively. For doing that, an IVT system was first set up by purifying both σ factors and the TFA core RNAP to reconstitute each RNAP holoenzyme. To know their functionality as σ factors, a titration of both EcfG1 and EcfG2 was assayed in IVT reactions using as template the promoter of *mltA2*. As shown in Supp. Fig. S3, the two forms of RNAP recognised and directed transcription from *P*_*mltA2*_ *in vitro*. However, transcription driven by EcfG2 was much higher than the one driven by EcfG1, which pointed out that EcfG2 has a greater potential to direct the transcription, at least with *mltA2* promoter.

**Figure 6:**
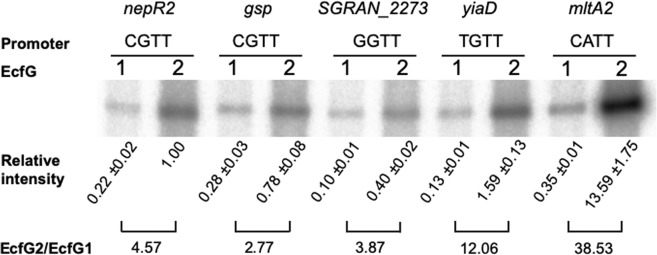
*In vitro* transcription from selected promoters representing most frequent GSR -10 boxes using either EcfG1 or EcfG2. The respective -10 boxes are indicated underneath each gene name. EcfG1 and EcfG2 are represented by 1 or 2, respectively. Values are represented in arbitrary units using the median intensity obtained for *P*_*nepR2*_ with EcfG2 as reference in the three independent replicates performed. Whole gels are shown in Supp. Fig. S4.

After this, in order to discard σ factor concentration effects, saturating concentrations of each EcfG protein were used in the subsequent IVT assays. Figure 6 shows the RNA amounts transcribed *in vitro* when equal amounts of either EcfG1 or EcfG2 (2.22 µM) were added to IVT reactions using the selected GSR promoter sequences as templates. As a first observation, the natural variability in the -35 box does not seem to affect the transcription in the tested promoters when comparing *nepR2* and *gsp*. The levels of RNA driven by EcfG1 were modest in absolute terms, but different transcript levels could be observed when comparing among them, reaching up to 5.11-fold compared to the reference (transcription obtained for *P*_*nepR2*_ using EcfG1). Transcription driven by EcfG2 was always higher than that mediated by EcfG1 regardless of the promoter used, again confirming its role as the master regulator of the GSR. However, a broader range of transcription levels was observed for EcfG2, reaching up to 13.59-fold compared to the reference (transcription obtained for *P*_*nepR2*_ using EcfG2). Thus, the EcfG2/EcfG1 transcription ratio ranged from 38.53-fold for *P*_*mltA2*_ to 2.77-fold for *P*_*gsp*_. Considering this, transcription driven by EcfG1 at some promoters may represent a considerable fraction of the transcriptional output when the transcription exerted by EcfG2 is only moderately higher than that obtained with EcfG1 (e.g., *P*_*gsp*_ *and P*_*SGRAN_2273*_).

This may explain why some promoters completely recover their transcription levels *in vivo* when EcfG1 is ectopically produced, whereas others need the presence of EcfG2 to be transcribed at a fully functional level. The cause of this, taking into account the function of a σ factor, might be either the capability of each protein to bind the promoter region or their efficiency to unwind the double strand to start the transcription^2^. Also, this phenomenon endorses that the relative contribution of each EcfG paralog depends on the characteristics of each promoter.

### Optimal transcription is driven when the -10 box matches the GSR consensus

**Figure 7:**
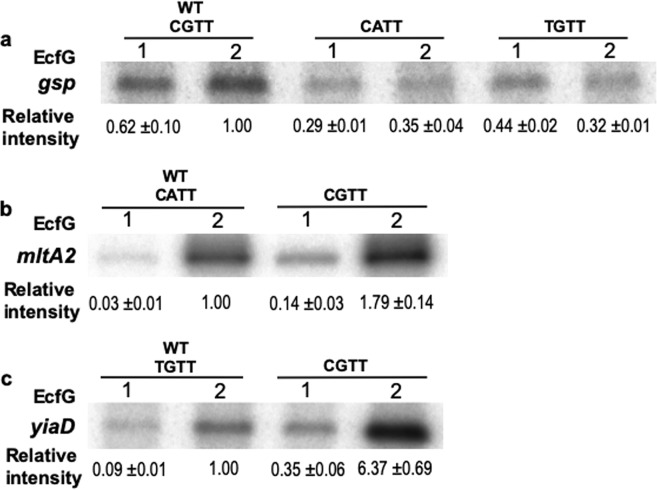
Effect of point mutations in the -10 box of *P*_*gsp*_, *P*_*mltA2*_ and *P*_*yiaD*_ promoters in IVT assays. Panels show the amounts of transcripts produced *in vitro* with EcfG1 or EcfG2 either from wild type or mutant versions of promoters *P*_*gsp*_ (**a**), *P*_*mltA2*_ (**b**) and *P*_*yiaD*_ (**c**). Values are represented in arbitrary units using the median intensity obtained for the respective wild type promoter with EcfG2 as reference in the three independent replicates performed. Due to the amount of transcripts obtained, phosphoscreens were exposed with the gels for approximately 15 min in the case of *P*_*mltA2*_ and 60 min in the case of *P*_*gsp*_ and *P*_*yiaD*_. Whole gels are shown in Supp. Fig. S5.

Given that the relative contribution of each EcfG protein to the transcription seems to be promoter-specific, special attention was focused on the -10 element. This sequence, as it was mentioned before, is less conserved among the GSR promoters than the -35 element. Considering that the -10 box plays a key role in the transcription initiation, and thus in the levels of transcription, the flexibility within that sequence might have been affecting the final output. In order to address this, IVT assays were performed with two types of mutant promoters: (i) mutating the CGTT consensus sequence in the *P*_*gsp*_ promoter to CATT or TGTT, and (ii) mutating the CATT and TGTT -10 boxes in the *P*_*mltA2*_ and *P*_*yiaD*_ promoters, respectively, to the consensus CGTT box. As shown in Fig. 7A a single point mutation diverging from the consensus -10 sequence decreased the transcription levels with respect to the wild type *P*_*gsp*_ promoter. Coherently with this result, a change toward the consensus -10 box in the *P*_*mltA2*_ and *P*_*yiaD*_ promoters clearly increased the transcription levels (Fig. 7B,C).

These results indicate that for each individual promoter, having a consensus -10 box would produce a maximum amount of transcripts. Considering the variability observed in that sequence among the GSR regulon, two interpretations may be drawn: firstly, having a non-consensus -10 box might be an intrinsic regulatory mechanism. Secondly, other features in the promoter region may contribute to the final output of the transcription, since promoters diverging from the consensus -10 box show higher levels of transcription than others bearing it. This strategy may allow each gene to be expressed at the level required for its physiological activity. In agreement with this observation, equal -35 and -10 surrounded by different spacer sequences between them and between the -10 box and the TSS produced diverse levels of transcription with both σ factors, but more pronouncedly with EcfG2.

Other studies have demonstrated that the properties of the spacer region between the -35 and -10 boxes affect the transcriptional output. For instance, for *E. coli* σ^70^ factor, the spatial conformation of the spacer region, even when altered in one nucleotide, may affect the transcription^37^. Another example of this can be found in *Bacillus subtilis*, where transcription led by ECFs σ^M^, σ^V^, σ^W^ and σ^X^ is modulated by a T-stretch that affects the spatial conformation of the spacer region^38^. In a scenario in which these four ECFs regulate overlapping sets of genes, the properties conferred by the T-stretch aid in the promoter selectivity, although it is not the only feature that allows the promoter specificity in that case^39,40^. Considering the variability in these spacer sequences among all the promoters within the GSR regulon described in TFA, the composition and the curvature in this region may explain the differences in the contribution of each EcfG protein to the transcription as a feature of each particular promoter. Even though we could not find a correlation among the genes that are better activated by EcfG1 and the ones that are more restricted to EcfG2 nor a functional specialisation between the two regulators, another possibility would be that the differentiation came from upstream in the signalling. Since there are two putative GSR regulatory elements (*nepR* and *phyR*) annotated in the TFA genome, the organisation of the pathway may define the EcfG protein that is predominantly active under certain conditions and thus, the subset of genes that is more highly expressed. Altogether, our results suggest that each gene in the GSR regulon presents a promoter sequence adapted to produce the appropriate level of transcription to exert its protective role in response to stress. The possible modulation exerted by the GSR regulatory elements upstream in the signalling cascade will be further investigated in future work.

## Conclusion

In this work, we show that *S. granuli* TFA responds to general stress by activating the transcription of 104 genes that are organised in 79 transcriptional units. We have identified the two σ factors governing this response, with EcfG2 being the master regulator, and EcfG1 acting as an accessory regulator. Even though EcfG1 may be dispensable to protect against stress, it is able to partially protect from adverse conditions when produced in sufficient amounts. The IVT assays of selected GSR-regulated genes have demonstrated that the intrinsic characteristics of each promoter determine the relative contribution of each EcfG protein to the transcriptional output, thus defining the differences between EcfG1 and EcfG2. These features lie mainly on the -10 box and the properties of the spacer region between the -35 and the -10 of each GSR-dependent promoter, suggesting that each gene has evolved to be expressed at the optimal level to exert its function in the protection against stress.

## Experimental procedures

### Media and growth conditions

*Escherichia coli* and *Sphingopyxis granuli* strains were routinely grown in LB rich medium at 37 °C or MML mineral medium at 30 °C, respectively. When indicated, *S. granuli* strains were grown in minimal medium supplemented with β-hydroxybutyrate, (BHB), as a carbon source in concentrations 8, 20 or 40 mM, depending on the experimental conditions.

### Plasmids, strains and oligonucleotides

Bacterial strains plasmids used in this work are described in Supp. Table S3. Oligonucleotides used for vector constructions, RT-qPCR quantifications and IVT vector constructions are listed in Supp. Table S4. For the generation of the *ecfG1* and *ecfG2* scarless deletions, the protocol described in^41^ was followed with some modifications. *ecfG1* upstream and downstream regions were amplified by PCR using oligonucleotide pairs del1161-1/del1161-2 and del1161-3/del1161-4, respectively. The resulting fragments were digested with BamHI and XhoI and ligated into pEMG cut with the same enzymes, resulting in plasmid pMPO1407. In the case of *ecfG2*, upstream and downstream regions were amplified using oligonucleotide pairs del1163-11/del1163-2 and del1163-3/del1163-4, respectively. The fragments were assembled together by overlapping PCR, digested with SacI and XbaI and cloned into pEMG cut the same to obtain pMPO1409. The same procedure was followed for the construction of pMPO1410, but using oligonucleotide del1163-12, with MPO855 chromosomic DNA as template instead of wild type DNA. As a first step for the deletion of *ecfG1* and *ecfG2*, pMPO1407 and pMPO1409, respectively, were transformed into TFA, and the single recombination event was selected selecting for kanamycin resistant colonies. Positive candidates were picked and transformed with pSW-I, and then selected for its resistance to ampicillin The second recombination event, which generates the in-frame deletion, was selected checking for both ampicillin-resistant and kanamycin-sensitive candidates, eventually resulting in the deletion mutants MPO855 and MPO859. The same protocol was used for MPO860 (Δ*ecfG1*Δ*ecfG2* double mutant) construction, using MPO855 as initial strain and pMPO1410 as vector.

For the generation of the *nepR2*, *gsp* and *ecfG2* translational fusions to *lacZ*, 1 kbp fragments upstream the ORF of each gene, including the promoter region and the first 5–8 codons, were amplified with oligo nucleotide pairs nepR2-lacZ fw/nepR2-lacZ rv, gsp-lacZ fw/gsp-lacZrv and del1163-11/del1163-2 (this last fragment was blunted with Klenow, New England Biolabs). These amplifications were clone in pJES379 digested with SmaI, resulting in vectors pMPO1408, pMPO1417 and pMPO1426, respectively. In order to monitor *in vivo* the expression of *nepR2* and *gsp* in the wild type and the mutant backgrounds, plasmids pMPO1408 and pMPO1417 were transformed in the wild type TFA, MPO855, MPO859 and MPO860, resulting in strains MPO858, MPO857, MPO863 and MPO864, respectively, for the *nepR2::lacZ* fusion, and MPO875, MPO876, MPO877 and MPO878, respectively, for the *gsp::lacZ* fusion. The same procedure was followed to introduce the *ecfG2::lacZ* fusion in the wild type TFA and MPO860, using pMPO1426, resulting in strains MPO891 and MPO892 respectively.

To construct a vector to complement the *ecfG2* mutation, a PCR fragment amplified with oligonucleotides ecfG2-1 EcoRI/ecfG2-2 BamHI and cut with EcoRI and BamHI was ligated into pLAFR3 digested with the same enzymes, generating pMPO1411. For the complementation of the *ecfG2* mutation by heterologous expression of e*cfG1*, plasmid pMPO1433, which carries *ecfG1* downstream the *Ptrc* promoter and its own Shine-Dalgarno sequence (that is, with the *nepR2* ORF removed from the *nepR2ecfG1*), was constructed. To do this, PCR fragments were amplified with primers nepR2 del1/nepR2 del2 and nepR2 del3/nepR2 del4, ensemble together by overlapping PCR, digested with XbaI and cloned into pMPO1412 cut with SmaI and XbaI, resulting in pMPO1413. Using this plasmid as template, a fragment was amplified using primers del1163-12/ecfG1-XbaI 5′, digested with SacI, blunted with T4 DNA polymerase (New England Biolabs) and digested with XbaI was ligated into pSEVA224 cut with HindIII, blunted with Klenow and digested cut with XbaI, obtaining pMPO1433, which carries *ecfG1* downstream its own Shine-Dalgarno sequence and *Ptrc* promoter.

To insert a 6xHis tag at the C-terminus end of the β′ subunit of the RNA polymerase for the purification of the whole core RNA polymerase, plasmid pMPO998 was constructed by cloning into vector pBluescript II KS a PCR fragment amplified with primers BetaB SacI/BetaB XbaI (the latter containing the 6xHis coding sequence) using TFA chromosomic DNA as template. Both fragment and vector were digested with SacI and XbaI and ligated. The resulting plasmid was introduced into the wild type TFA and inserted into the chromosome by recombination, obtaining strain MPO700.

For overexpression prior to purification of EcfG1 and EcfG2, pMPO1431 and pMPO1432, respectively, were constructed following the IMPACT kit’s manual (New England Biolabs). PCR fragments were amplified using ORF-ecfG1 fw/ORF-ecfG1 rv BamHI and ORF-ecfG2 fw/ORF-ecfG2 rv BamHI respectively, digested with BamHI and cloned into the expression vector pTYB21 digested with SapI, blunted with Klenow and digested again with BamHI.

For the construction of *in vitro* transcription template vectors (pMPO1440–1444, pMPO1447–1450), primers containing both strands (sense and antisense, see Supp. Table S4) of selected promoter regions and compatible ends for ligation into EcoRI and HindIII sites were synthesised. Respective sense and antisense primers were annealed in stoichiometric proportions by means of a temperature ramp in a thermocycler (from 95 °C to 25 °C) and cloned into pTE103 digested with EcoRI and HindIII.

All plasmid constructions were confirmed by sequencing and strains were checked either by PCR or by Southern blotting.

### Stress phenotypic assays

To test the resistance to osmotic stress and copper, 10 μl spots of serial dilutions of late-exponential phase cultures were placed on solid MML rich medium plates supplemented with NaCl 0.6 M or CuSO_4_ 3.5 mM and incubated for 5 days at 30 °C. For desiccation assays, 5 μl spots of serial dilutions of late-exponential phase cultures were placed on 0.45 μm pore size filters (Sartorius Stedim Biotech GmbH, Germany) and they left to air-dry in a laminar flow cabin for 5 h (5 min in the control assay). Then, filters were placed on MML rich medium plates supplemented with bromophenol blue 0.002% and incubated for 5 days at 30 °C. In the case of resistance to oxidative shock, late-exponential phase cultures were diluted to an OD_600_ of 0.1. When an OD_600_ of 0.5 was reached, H_2_O_2_ was added to the medium up to a concentration of 10 mM. Recovery from the shock is represented by a percentage of the OD_600_ reached by treated cultures after 5 h of growth compared to non-treated cultures. At least three independent replicates of each experiment were performed, and most representative examples are shown.

### GSR activation assays and expression measurements

Saturated preinocula were diluted to an OD_600_ of 0.05 in minimal medium supplemented with β-hydroxybutyrate 40 mM and incubated at 30 °C in an orbital shaker for 16 h. Then, 20 ml of minimal medium with β-hydroxybutyrate 8 mM were inoculated at OD_600_ 0.1 of the strain to be assayed. In the case of strains ectopically expressing *ecfG1*, IPTG 1 mM was added from the beginning of the growth. 1 ml aliquots were withdrawn 72 h after starting the culture to measure growth and GSR activation by β-galactosidase activity from *nepR2*. Averages of three independent replicates are represented. To measure the expression from the *ecfG2* promoter, the procedure mentioned above was followed, but samples were taken at different time points since the expression did not change over time.

### Total RNA extraction

Late-exponential phase cultures grown in minimal medium with β-hydroxybutyrate 40 mM were diluted to an OD_600_ of 0.1 in minimal medium supplemented with β-hydroxybutyrate 20 mM and incubated at 30 °C in an orbital shaker for 30 h. 10 ml of each culture were centrifuged and the pellets were snap-frozen in liquid nitrogen. Total RNA purification was carried out according to the protocol previously described^42^ from the different strains at the beginning of the stationary phase. DNA was removed by DNase I treatment (DNA-free kit, Ambion, Inc.). Once the DNA removal was confirmed by PCR amplification, RNA samples were purified using the RNeasy purification kit (Qiagen, Germany). RNA integrity was checked by agarose gel electrophoresis.

### dRNA-seq analysis

Equimolecular amounts of three independent RNA extraction replicates were mixed for the wild type TFA strain and strain MPO860. Samples were sent to the company ASCIDEA (http://www.ascidea.com/lifesciences-services.html) for cDNA library preparation and high-throughput sequencing using an Illumina HiSeq2000 machine.

### dRNA-seq bioinformatic analysis

The normalization and the analyses of the dRNA-seq results were performed by ASCIDEA. In summary, quality of the reads obtained by HiSeq2000 sequencing was checked with FastQC software FASTX-Toolkit (http://hannonlab.cshl.edu/fastx_toolkit/index.html) and ASCIDEA specific perl scripts were run as low quality region filters. Adaptors and low quality bases at the ends of sequences and reads with undetermined bases or with 80% of their bases with less than 20% quality score were trimmed. Reads that passed these filters were mapped using^43^ to generate read alignments using the *Sphingopyxis granuli* strain TFA genome (RefSeq: NZ_CP012199.1) as ref. ^18^. Differential transcript expression was then computed using DESeq2. Genes downregulated more than 3-fold comparing strain MPO860 to TFA were selected for further analysis.

### Motif search and sequence analysis

450 bp sequences upstream the start codon of genes more than 3-fold downregulated in MPO860 compared to the wild type TFA were subjected to motif search using the online tool MEME^25^. The significant parameters considered in the analysis were: motif occurrences: 0 or 1; number of different motifs: 5; minimum and maximum motif width: 23–28; searching only in the given strand. Consensus sequence retrieved by MEME was thrown back to the pool of sequences using FIMO^44^ in order to detect low quality matches. Resulting sequences were manually curated and refined according to the distance of the putative consensus to possible transcription start sites previously described in this strain^18^ or to the respective gene start codon, the similarity of the putative -35 and -10 boxes to the overall TFA consensus and the length of the spacer region between the putative -35 and -10 boxes. Also, the putative operon organization of the regulated genes was analysed according to the expression data shown in this work and in previously reported data^18^ by visualizing the using the Integrated Genome Browser (IGB).

### RT-qPCR analyses

To validate the data obtained from the dRNA-seq, total RNA was extracted from three independent replicates of strains TFA, MPO855, MPO859, and MPO860 in the same conditions performed for the dRNA-seq experiment (see above). cDNA samples were generated using the High-Capacity cDNA Archive Kit (Applied Biosystems) and purified with the QIAquick PCR purification kit (Ambion). RT-qPCR experiments were performed using the universal kit FastGene ICGreen (Nippon Genetics Europe GmbH, Germany) and the oligonucleotide pairs listed in Supp. Table S4 according to the manufacturer’s instructions. Reactions were run in a CFX Connec Real-Time PCR Detection System (BioRad) in triplicates.

### Protein overexpression and purification

*S. granuli* core RNA polymerase purification was based on Burgess protocol^45^ with modifications from Hager^46^ and Patek^47^. Saturated cultures of *S. granuli* MPO700 grown in MML were diluted to OD_600_ 0.1 in 1 L of MML. The culture was incubated at 30 °C and 180 rpm up to OD_600_ 0.6–0.9. Then, the grown cells were collected by centrifugation 8.000 *g* 20 min at 4 °C, and the pellet was kept at −80 °C. A total of 22.5 OD_600_ unit were used for the purification. The biomass was resuspended in 200 mL of grinding buffer (Tris-HCl 50 mM pH 8, EDTA 2 mM, glycerol 5% (v/v), NaCl 0.2 M, DTT 0.1 mM, β-mercaptoethanol 1 mM, PMSF 0.1 mM) and broken by sonification (15 min, 2 seg off, 2 seg on, 30% amplitude). The cell extract was centrifuged during an hour at 13.000 *g* 4 °C. The soluble fraction was fractioned with 0.35% Polimin P pH 7.9 (Sigma). The mixture was incubated 15 min with shaking at 4 °C and then pelleted during 20 min at 7630 *g* 4 °C. The supernatant was discarded and the pellet was resuspended in 150 mL of cold TGED (Tris-HCl 10 mM pH 8, EDTA 0.1 mM, glycerol 10% (v/v), DTT 0.1 mM) with NaCl 0.5 M and centrifuged 20 min at 7630 *g* 4 °C. This step was repeated twice. The pellet was resuspended in 100 mL of cold TGED NaCl 1 M and two tablets of proteases inhibitor (Complete Protease Inhibitor Cocktail Tablets, Roche). The mixtures were stirred during 30 min at 4 °C and centrifuged 30 min at 4290 *g* 4 °C. The supernatant was precipitated overnight at 4 °C using 35.5 g of ammonium sulphate. After that, the sample was centrifuged 30 min at 7630 *g* 4 °C and the supernatant was discarded. The sediment was resuspended in 50 mL of cold TGED and two tablets of proteases inhibitor. The mixture was centrifuged 20 min at 7630 *g* 4 °C and the supernatant was dialysed at 4 °C to reduce salinity using cellulose membranes (33 mm of pore diameter). The dialyses was performed in 4 steps using 2 L of buffer P (Na_2_HPO_4_ 50 mM, glycerol 5% (v/v), 3 mM β-mercaptoethanol, PMSF 0,1 mM pH 8) with decreasing amount of salt, from NaCl 0.5 M to NaCl 0.3 M with imidazol 10 mM. The last step of dialysis was performed overnight. The desalted sample was incubated overnight at 4 °C with 5 mL of nickel agarose beads (High Density Cobalt, ABT) previously equilibrated in buffer P NaCl 0.3 M imidazol 10 mM. The mixture was decanted by gravity using a Glass Econo-Column (BioRad) and the resin was washed with 50 mL of buffer P with NaCl 300 mM imidazole 10 mM preincubated 20 min. This step was performed twice. The next wash was done with 25 mL of the same buffer but with 20 mM of imidazole. The washing was eluted by gravity. Finally, the resin was incubated 30 min with 5 mL of buffer P NaCl 0.3 M imidazol 0.5 M and was eluted by gravity. A second elution was performed with 3 mL of the same buffer. The eluted fractions were analysed by SDS-PAGE and concentrated using 10 KDa centricons (Millipore Amicon Ultra-15 Centifugal Filter Concentrator). Simultaneously, the buffer was change to TGED NaCl 100 mM and finally conserved in 50% of glycerol at −80 °C.

EcfG1 and EcfG2 proteins were purified using the IMPACT kit (New England Biolabs) following the manufacturer’s instructions. Briefly, pMPO1431 and pMPO1432 (for overexpression of *ecfG1* and *ecfG2* respectively) were transformed into *E. coli* strain ER2566. Saturated preinocula of each strain were diluted to an OD_600_ of 0.1 in 400 ml of LB medium and incubated at 37 °C in an orbital shaker until OD_600_ 0.5 was reached. Then, cultures were ice-cooled for 30 min. After that, cultures were induced with IPTG 0.5 mM and incubated at 14 °C in a shaker for 16 h. Cultures were centrifuged and the induction was confirmed by SDS-PAGE. Cells were resuspended in binding buffer (Tris-HCl 20 mM pH 8, NaCl 0.5 M) and mechanically lysed by sonication. Lysates were clarified by centrifugation. Once the chitin resin was packed in a purification column and washed with binding buffer, the respective clarified lysates were loaded on the column and left to flow through the resin by gravity. The column was flushed with 100 ml of binding buffer prior to the induction of the on-column protein cleavage. To release the target protein, the resin was incubated with binding buffer supplemented with DTT 50 mM at 18 °C for 40 h approximately. The proteins were eluted and the enrichment of the target protein was confirmed by SDS-PAGE. The eluate was dialysed against modified TEDG buffer 2X(Tris-HCl 100 mM pH 8, glycerol 10%, Triton X-100 0.02%, EDTA 0.2 mM, NaCl 100 mM, DTT 0.2 mM) at 4 °C overnight. Protein solutions were concentrated to a volume of approximately 300 μl using Amicon Ultra centrifugal filters (Millipore) of different pore size. Then samples were diluted with 1 vol of glycerol 99% to a final glycerol concentration of 50%. After protein quantification (RC DC Protein Assay kit, BioRad), protein aliquots were conserved at −80 °C.

### *In vitro* transcription

Multi-round *in vitro* transcription reactions (adapted from Porrúa *et al*.^48^) were run in a final volume of 22.5 μl *in vitro* transcription buffer (Tris-HCl 10 mM pH8, NaCl 50 mM, MgCl_2_ 5 mM, KCl 100 mM, BSA 0.2 mg/ml, DTT 2 μM) at 30 °C. RNA core polymerase was preincubated at 30 °C for 5 min, then the appropriate amount of EcfG σ factor diluted in TEDG buffer without glycerol was added to the reaction mixtures and incubated for other 5 min. Then 0.5 μg of the appropriate plasmid were added as circular template. 5 min later, a mix of ATP, GTP, CTP (final concentration of 0.4 mM), UTP (0.07 mM) and [α−32P]-UTP (0.33 mM, Perkin Elmer) was added start the reaction. After 10 min, reaction reinitiation was prevented by adding heparin to a final concentration of 0.1 mg/ml, and 10 min later reactions were stopped by adding 5 μl of stop/loading buffer (0.5% formamide, 20 mM EDTA, 0.05% bromophenol blue, 0.05% xylene cyanol). Samples were boiled for 3 min and run on 4% polyacrylamide-urea denaturing gels in Tris-borate-EDTA buffer at room temperature. Gels were dried and exposed in a phosphoscreen. Results were visualised in an Amersham Typhoon scanner and analysed using the ImageQuant software (both provided by GE Healthcare Bio-Sciences AB).

## Supplementary information

Supplementary information.

Supplementary information2.

Supplementary information3.
