# Supplementary material for: Two paralogous EcfG σ factors hierarchically orchestrate the activation of the General Stress Response in Sphingopyxis granuli TFA
Source: Sci Rep. 2020 Mar 20;10:5177. doi: 10.1038/s41598-020-62101-z (PMC7083833; doi:10.1038/s41598-020-62101-z)
Supplement: Supplementary file 3 — Supplementary information3. [file 41598_2020_62101_MOESM3_ESM.pdf]

SUPPLEMENTARY INFORMATION

Two paralogous EcfG σ factors hierarchically orchestrate the activation of the General Stress Response in *Sphingopyxis granuli* TFA.

Rubén de Dios, Elena Rivas-Marín, Eduardo Santero and Francisca Reyes-Ramírez*.
Centro Andaluz de Biología del Desarrollo, Universidad Pablo de Olavide/Consejo Superior de Investigaciones Científicas/Junta de Andalucía and Departamento de Biología Molecular e Ingeniería Bioquímica, Universidad Pablo de Olavide.

Supplementary Figure 1. Absolute frequency of the genes ≥3-fold up- or downregulated in the Δ*ecfG1*Δ*ecfG2* double mutant with respect to the wild type TFA regarding their putative function according to the Cluster of Orthologous Genes (COG) broad classification.

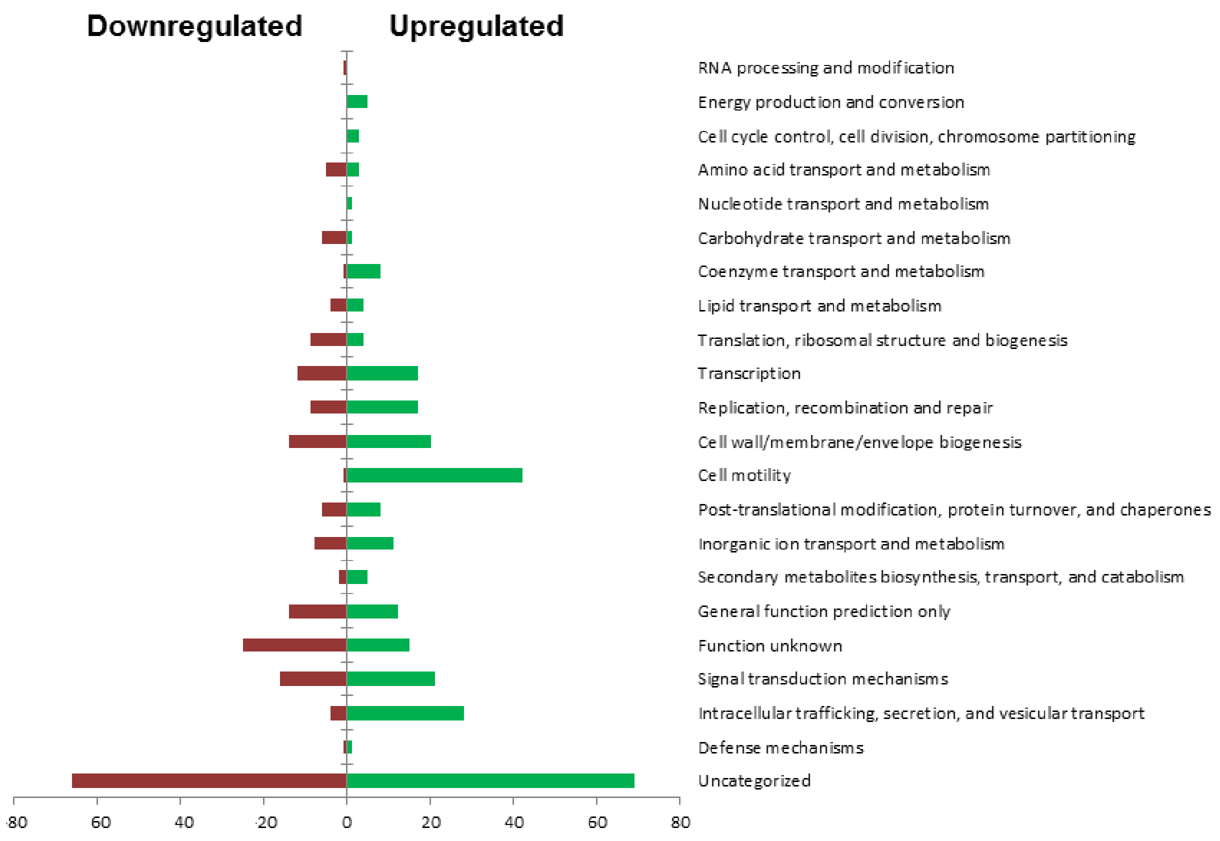

Supplementary Figure 2. Absolute frequency of the genes belonging to the GSR regulon in *S. granuli* TFA regarding their putative function according to the Cluster of Orthologous Genes (COG) broad classification.

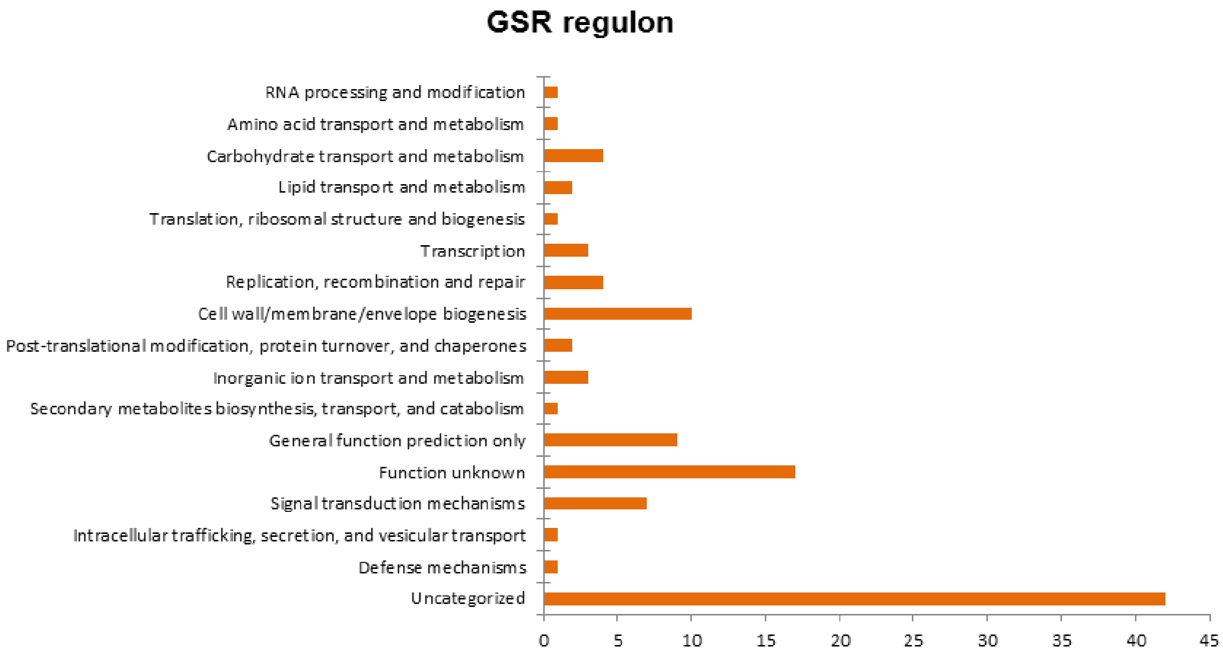

Supplementary Figure 3. Titration of EcfG1 (a) and EcfG2 (b) using the P_{mltA2} promoter as template. Both σ factors were added to the reaction at a final concentration of 0, 0.44, 0.89, 1.33, 1.78 and 2.22 µM. Arbitrary units were calculated using the median intensity obtained with the maximum EcfG concentration as reference in three independent replicates. Whole gel showing the results of the titration is shown in panel (c). Lanes correspond to: A1-6) P_{mltA2} with increasing concentrations of EcfG2 (0, 0.44, 0.89, 1.33, 1.78 and 2.22 µM); B1-6) P_{mltA2} with increasing concentrations of EcfG1 (0, 0.44, 0.89, 1.33, 1.78 and 2.22 µM).

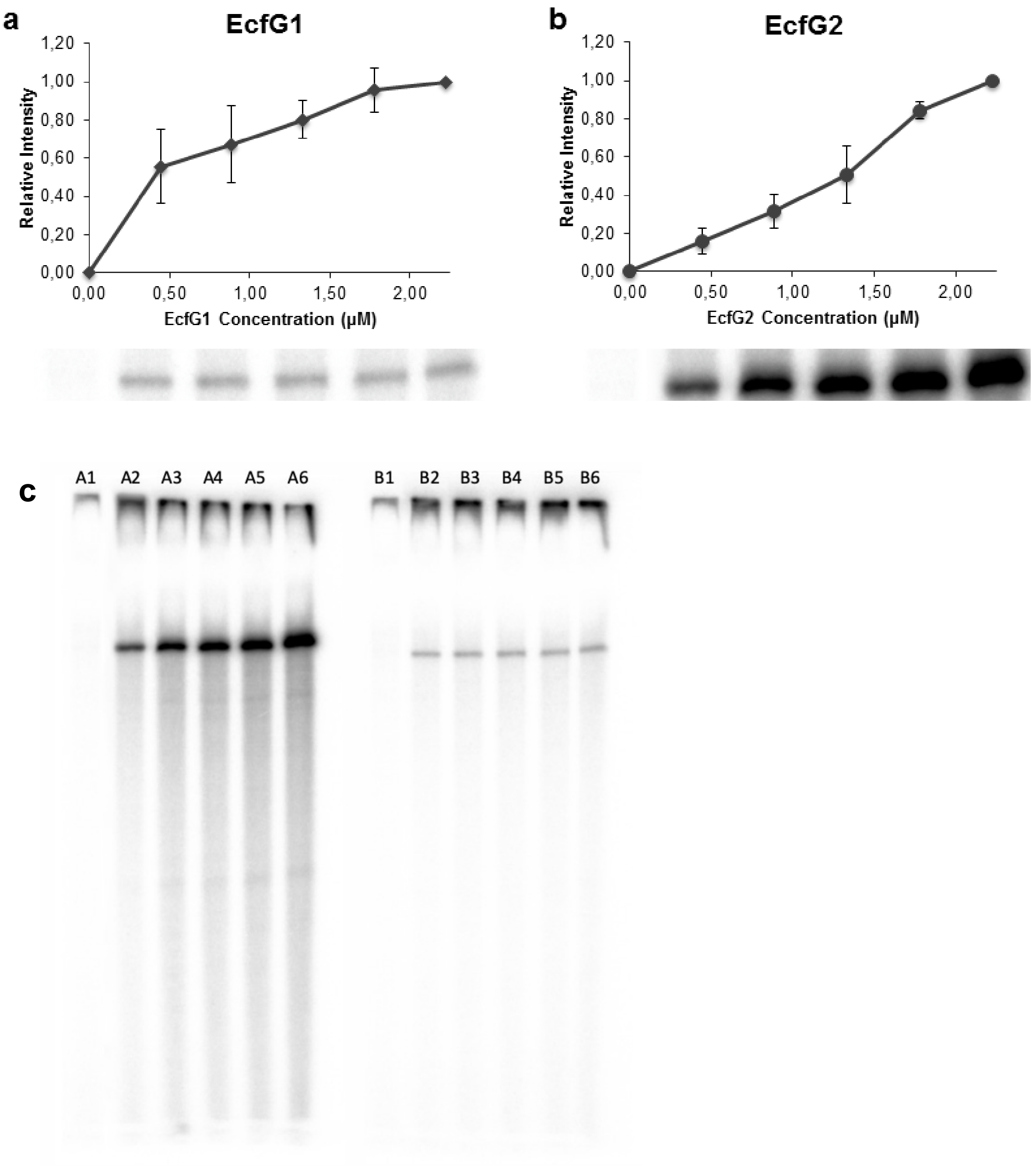

Supplementary Figure S4. Whole gel containing the results exposed in Figure 6, showing the transcription obtained with different promoter using equal amounts of EcfG1 or EcfG2. Lanes correspond to: A1) P_{nepR2} with EcfG1; A2) P_{nepR2} with EcfG2; B1) P_{gsp} with EcfG1; B2) P_{gsp} with EcfG2; C1) P_{SGRAN_2273} with EcfG1; C2) P_{SGRAN_2273} with EcfG2; D1) P_{yiaD} with EcfG1; D2) P_{yiaD} with EcfG2; E1) P_{mltA2} with EcfG1; E2) P_{mltA2} with EcfG2.

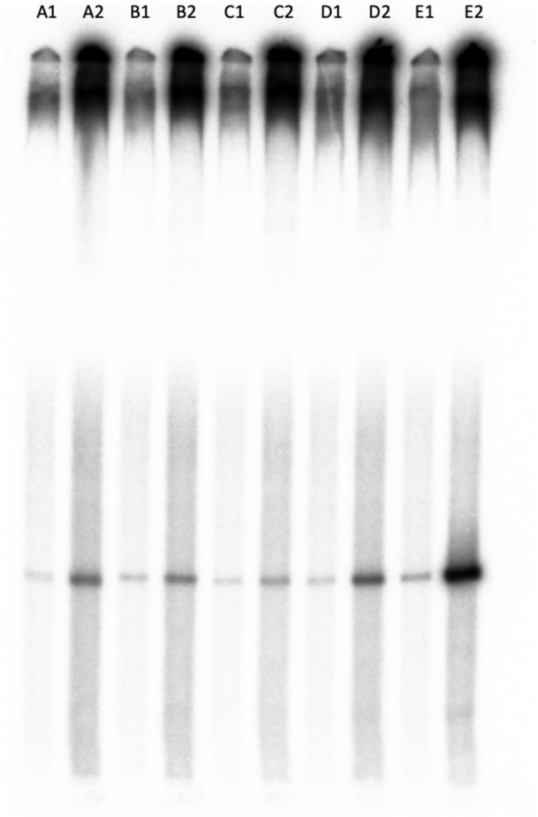

Supplementary Figure S5. Whole gel containing the results exposed in Figure 7, showing the transcription obtained with different mutated promoter versions. a) P_{gsp} (lanes correspond to: A1) P_{gps}-wild type with EcfG1; A2) P_{gsp}-wild type with EcfG2; B1) P_{gsp}-CATT with EcfG1; B2) P_{gsp}-CATT with EcfG2; C1) P_{gsp}-TGTT with EcfG1; C2) P_{gsp}-TGTT with EcfG2). b) P_{mltA2} (lanes correspond to: A1) P_{mltA2}-wild type with EcfG1; A2) P_{mltA2}-wild type with EcfG2; B1) P_{mltA2}-CGTT with EcfG1; B2) P_{mltA2}-CGTT with EcfG2; first four lanes from left to right correspond to an experiment unrelated to this manuscript). c) P_{yiaD} (lanes correspond to: A1) P_{yiaD}-wild type with EcfG1; A2) P_{yiaD}-wild type with EcfG2; B1) P_{yiaD}-CGTT with EcfG1; B2) P_{yiaD}-CGTT with EcfG2) using equal amounts of EcfG1 or EcfG2.

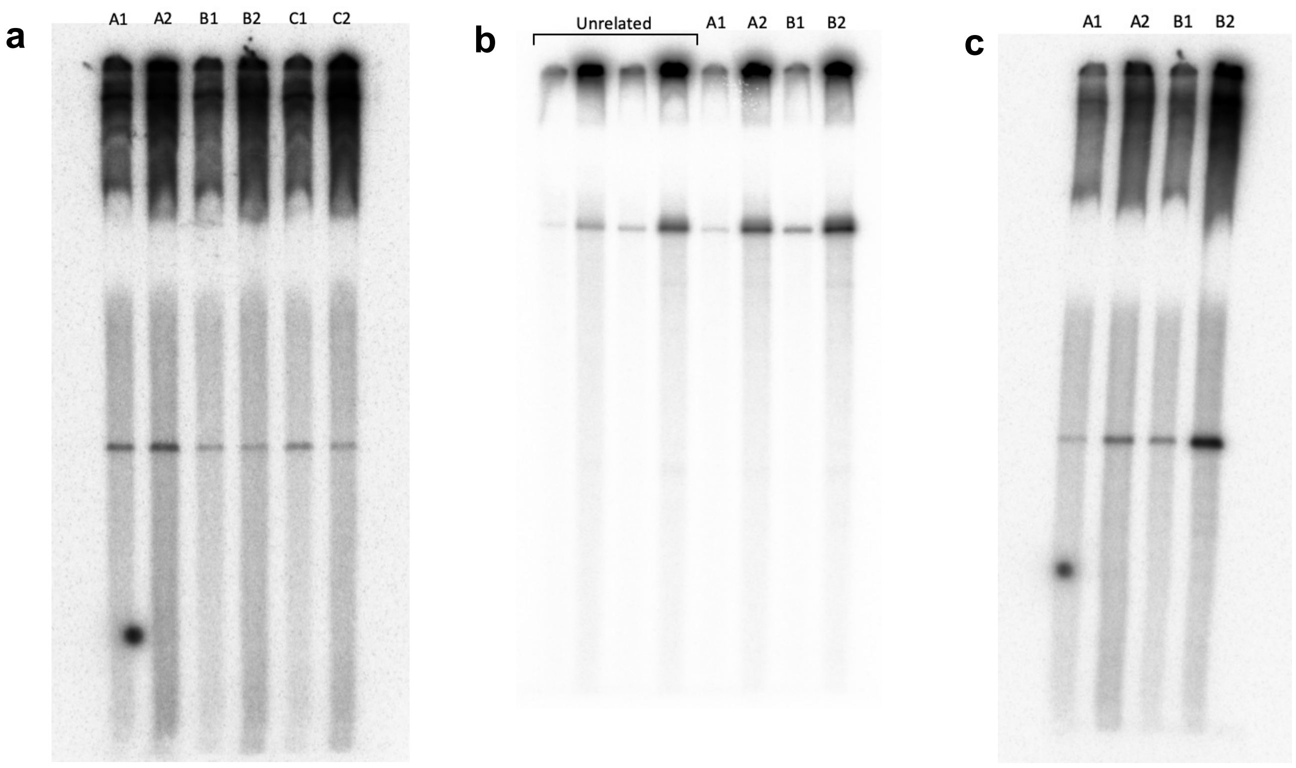

Supplementary Table S1 (provided as excel file). Genes upregulated and downregulated (in pages 1 and 2 of this document, respectively) more than 3-fold in the Δ*ecfG1*Δ*ecfG2* mutant compared to the wild type TFA. The relative expression, the genome strand where the genes are encoded, the COG functional category [1] where they belong (the category represented by each symbol is shown in page 3 of this document), the annotated gene name and the putative function are shown in successive columns.

Supplementary Table S2 (provided as excel file). Genes defined within the GSR regulon in *S. granuli* TFA. The relative reduction (and increase) in their expression are shown in columns B and C, respectively. The genome strand where they are encoded, the COG functional category [1] where they belong (the category represented by each symbol is shown in page 2 of this document), their annotated gene name and their putative function are shown in figures D-G. The promoter sequence containing the putative -35 and -10 boxes are shown in column H (-35 and -10 boxes are represented in bold), and when genes are organised in a putative operon it is indicated in column I.

Supplementary Table S3. Strains and plasmids used in this work.

**Strain**	**Relevant features**	**Reference**
	Escherichia coli	
DH5α	F$^-$ *φ80lacZΔM15, Δ(lacZYA-argF)U169, recA1, endA1, hsdR17(rK-m K*$^-$*), supE44, thi-1, gyrA, relA1*	Hanahan *et al.* [2]
DH5α/*λpir*	F$^-$ *φ80lacZΔM15, Δ(lacZYA-argF)U169, recA1, endA1, hsdR17(rK-m K*$^-$*), supE44, thi-1, gyrA, relA1, λpir* lysogen	Martinez-Garcia & de Lorenzo[3]
ER2566	F$^-$ λ$^-$ *fhuA2, [lon], ompT, lacZ::T7p07, gal, sulA11, Δ(mcrC-mrr)114::IS10, R(mcr-73::miniTn10-TetS)2, R(zgb-210::Tn10)(TetS), endA1, [dcm]*	New England Biolabs
	Sphingopyxis granuli	
TFA	Wild type strain, Str^r	Hernaez *et al.* [4]
MPO700	wild type TFA; *rpoC::6xHis* by pMPO998 integration in the chromosome, Ap^r	This work
MPO855	*ΔecfG1* (*ΔSGRAN_1161*), Str^r	This work
MPO857	*ΔecfG1* (*ΔSGRAN_1161*); *nepR2::lacZ* by pMPO1408 integration in the chromosome, Ap^r	This work
MPO858	wild type TFA; *nepR2::lacZ* by pMPO1408 integration in the chromosome, Ap^r	This work
MPO859	*ΔecfG2* (*ΔSGRAN_1163*), Str^r	This work
MPO860	*ΔecfG1ΔecfG2* (*ΔSGRAN_1161ΔSGRAN_1163*), Str^r	This work
MPO863	*ΔecfG2* (*ΔSGRAN_1163*); *nepR2::lacZ* by pMPO1408 integration in the chromosome, Ap^r	This work
MPO864	*ΔecfG1ΔecfG2* (*ΔSGRAN_1161ΔSGRAN_1163*); *nepR2::lacZ* by pMPO1408 integration in the chromosome, Ap^r	This work
MPO891	wt TFA; *ecfG2::lacZ* by pMPO1426 integration in the chromosome, Ap^r	This work
MPO892	*ΔecfG1ΔecfG2* (*ΔSGRAN_1161ΔSGRAN_1163*); *ecfG2::lacZ* by pMPO1426 integration in the chromosome, Ap^r	This work
**Plasmids**	**Relevant features**	**Reference**
pBluescript II KS	Cloning vector, Ap^r	Stratagene
pEMG	oriR6K, *lacZα* with two flanking I-SceI restriction sites, Km^r	Martinez-Garcia & de Lorenzo[3]
pJES379	Vector for translational fusions to *lacZ*, Ap^r	Santero *et al.*[5]
pMPO998	pBluescript derivative plasmid containing the C-terminus end of the TFA *rpoC* gene fused to a 6xHis tag, Ap^r	This work
pMPO1407	pEMG derivative bearing *ecfG1* flanking regions for its deletion, Km^r	This work
pMPO1408	pJES379 derivative bearing a *nepR2::lacZ* translational fusion, Ap^r	This work
pMPO1409	pEMG derivative bearing *ecfG2* flanking regions for its deletion in a wild type background, Km^r	This work
pMPO1410	pEMG derivative bearing *ecfG2* flanking regions for its deletion in a Δ*ecfG1* mutant background, Km^r	This work
pMPO1412	pEMG derivative with *rpsL1* streptomycin counterselection marker, Km^r	Gonzalez-Flores *et al.*[6]

pMPO1413	pMPO1412 derivative bearing the *nepR2ecfG1* operon with an in-frame deletion in *nepR2*, Km^r	This work
pMPO1426	pJES379 derivative bearing a *ecfG2::lacZ* translational fusion, Ap^r	This work
pMPO1431	pTYB21 derivative bearing *intein::ecfG1* fusion for EcfG1 purification, Ap^r	This work
pMPO1432	pTYB21 derivative bearing *intein::ecfG2* fusion for EcfG2 purification, Ap^r	This work
pMPO1433	pSEVA224 derivative bearing *ecfG1* expressed under P_{trc} promoter, Km^r	This work
pMPO1440	pTE103 derivative for *nepR2 in vitro* transcription, Ap^r	This work
pMPO1441	pTE103 derivative for *gsp in vitro* transcription, Ap^r	This work
pMPO1442	pTE103 derivative for *mltA2 in vitro* transcription, Ap^r	This work
pMPO1443	pTE103 derivative for *yiaD in vitro* transcription, Ap^r	This work
pMPO1444	pTE103 derivative for *SGRAN_2273 in vitro* transcription	This work
pMPO1447	pTE103 derivative for *mltA2 in vitro* transcription, the -10 box is mutated to CGTT, Ap^r	This work
pMPO1448	pTE103 derivative for *yiaD in vitro* transcription, the -10 box is mutated to CGTT, Ap^r	This work
pMPO1449	pTE103 derivative for *gsp in vitro* transcription, the -10 box is mutated to CATT, Ap^r	This work
pMPO1450	pTE103 derivative for *gsp in vitro* transcription, the -10 box is mutated to TGTT, Ap^r	This work
pSEVA224	oriRK2, standard polylinker, *$lacI^q$-P_{trc}*, Km^r	Silva-Rocha *et al.*[7]
pSW-I	oriRK2, xylS, *P_m::I-sceI*, Ap^r	Martinez-Garcia & de Lorenzo[3]
pTE103	Apr, transcription template vector with T7 terminator downstream of pUC8 polylinker, Ap^r	Elliott & Geiduschek[8]
pTYB21	*E. coli* expression vector, bears an N-terminus fusion intein variant for purification of the target protein by IMPACT kit, Ap^r	New England Biolabs

Supplementary Table S4. Oligonucleotides used in this work.

**Olignucleotide**	**Sequence**
**Construction primers**	
BetaB SacI	CAAGAAGGAAGAGCTCCAGC
BetaB XbaI	ATGCTATCTAGATTAGTGGTGGTGGTGGTGGTGTTCTTCCTCGCCG
del1161-1	CGGGATCCACCAGATTGACCTCG
del1161-2	CGCTCGAGCCGTGTTCCCGCAC
del1161-3	GGCTCGAGTCATTCCTTGGTCG
del1161-4	CAGGATCCAATTGCGCAGGATG
del1163-11	TGAGCTCCAGGATGACGAAGGTCC
del1163-12	TGAGCTCTCATCATCGCAAATACTCG
del1163-2	AAACTGGCGTCGTCGAG
del1163-3	CCGCTCGACGACGCCAGTTTCGATCTGACTGCGAACG
del1163-4	TTTCTAGACCTTGCTGGAAGTCATCG
ecfG1-XbaI 5'	TATCTAGAGAATGGCAACGCACGGAG
ecfG2-1 EcoRI	ATGAATTCGGTGCAGCGGGTAGCATC
ecfG2-2 BamHI	TAGGATCCGAACCTATCATGCCGCTTC
gsp-lacZ fw	CGGGATATGATGCGCGTG
gsp-lacZ rv	GGGCGATCAGGATGAAGG
nepR2-lacZ fw	GATGTTGCCATAGATCATGCG
nepR2-lacZ rv	GGCCGCTAGCTCGTCGTAG
nepR2 del1	GGCGCGAGGATGACGTC
nepR2 del2	AAGGAATGACCCGAACCC
nepR2 del3	CTTGGGTTCGGGTCATTCCTTAAAGCCACCGTGCATCCG
nepR2 del4	TATCTAGACGGCACCCTCGTCATAGG
ORF-ecfG1 fw	ATGACCCGAACCCAAGCTGC
ORF-ecfG1 rv BamHI	TTGGATCCTCAGCGCCGCGATTCCTC
ORF-ecfG2 fw	ATGAGCCGCACCTCCGAC
ORF-ecfG2 rv BamHI	TTGGATCCCCATACGGCATATTCACCCC
**RT-qPCR primers**	
ecfG2-Q1	CTCGCCCTGAGCAAAATCG
ecfG2-Q2	TCGCACCGGTGGCAAATAC
csbD-Q1	TGCGGCCTCGATCAACAAG
csbD-Q2	GACCGCCTGCTTCGCATTG
gsp-Q1	CGATATCAAGCAGGATTTCTG
gsp-Q2	TGCGCATTCATCGGGATATG
mltA2-Q1	CCGAACCGACAGAGAAAGG
mltA2-Q2	GACGCCGACGACGATGTTG
nepR2-Q1	TCTGATCGAGCTGGGACAAG
nepR2-Q2	GACGGGAAGCGACCAGTG
phyR2-Q1.2	CCATTGGGCGAAGAAATCC
phyR2-Q2	ATGACTTCCAGCAAGGTATG

SGRAN_1165-Q1	TTGATTTGGGAACCGGATTC
SGRAN_1165-Q2	TCCCCACCGTCGGCCAATG
SGRAN_1766-Q1	GGTGACGATCCCTTCAAGTC
SGRAN_1766-Q2	TGAAGGGCAAGGAGGCTATC
SGRAN_1922-Q1	GACGCGTAACCGTGCTATC
SGRAN_1922-Q2	GACCCCTTTCACCGTATTGC
SGRAN_2195-Q1	ATGGCCGACACAATCGAAAG
SGRAN_2195-Q2	TCGTCCTGCGGGTTGTAGAC
SGRAN_2273-Q1	GCCGCGGGTTGTCGAATCC
SGRAN_2273-Q2	GCGCCGATCAAAAGCATATG
SGRAN_4136-Q1	GATCCCCGTCGAAATCTATC
SGRAN_4136-Q2	ACGACCTTCTCATATTTAACC
yiaD-Q1	CGATCAAGCTCACCATGTTC
yiaD-Q2	GATCGCGGCCTTGGAAATG
**IVT construction primers**	
gsp-IVT fw	AATTGCAACATTGCCGCCGCTGCCGCGTTGATCGGTCATCGGG
gsp-IVT rv	AGCTCCCGATGACCGATCAACGCGGCAGCGGCGGCAATGTTGC
gsp-IVT-CATT fw	AATTGCAACATTGCCGCCGCTGCCGCATTGATCGGTCATCGGG
gsp-IVT-CATT rv	AGCTCCCGATGACCGATCAATGCGGCAGCGGCGGCAATGTTGC
gsp-IVT-TGTT fw	AATTGCAACATTGCCGCCGCTGCCGTGTTGATCGGTCATCGGG
gsp-IVT-TGTT rv	AGCTCCCGATGACCGATCAACACGGCAGCGGCGGCAATGTTGC
mltA2-IVT fw	AATTGGAACCTTTTTCGCCCTTCTCGCATTATGTCCGCATCGTC
mltA2-IVT rv	AGCTGACGATGCGGACATAATGCGAGAAGGGCGAAAAAGGTTCC
mltA2-IVT-CGTT fw	AATTGGAACCTTTTTCGCCCTTCTCGCGTTATGTCCGCATCGTC
mltA2-IVT-CGTT rv	AGCTGACGATGCGGACATAACGCGAGAAGGGCGAAAAAGGTTCC
nepR2-IVT fw	AATTGGAACCTGCGGCCCCCTCTGTCGTTGCTTCGGAATGGCA
nepR2-IVT rv	AGCTTGCCATTCCGAAGCAACGACAGAGGGGGCCGCAGGTTCC
SGRAN_2273-IVT fw	AATTGCAACTTTCGCGGCGCGCGGGCGGTTGTTCTCATGCCCCT
SGRAN_2273-IVT rv	AGCTAGGGGCATGAGAACAACCGCCCGCGCGCCGCGAAAGTTGC
yiaD-IVT fw	AATTGGAACCAGATGCGCGGCCATATGTTATCGCCATGGAGGG
yiaD-IVT rv	AGCTCCCTCCATGGCGATAACATATGGCCGCGCATCTGGTTCC
yiaD-IVT-CGTT fw	AATTGGAACCAGATGCGCGGCCATACGTTATCGCCATGGAGGG
yiaD-IVT-CGTT rv	AGCTCCCTCCATGGCGATAACGTATGGCCGCGCATCTGGTTCC

Supplementary References

1 Tatusov, R. L., Galperin, M. Y., Natale, D. A. & Koonin, E. V. The COG database: a tool for genome-scale analysis of protein functions and evolution. *Nucleic Acids Res* **28**, 33-36, doi:10.1093/nar/28.1.33 (2000).

2 Hanahan, D. Studies on transformation of Escherichia coli with plasmids. *J Mol Biol* **166**, 557-580, doi:10.1016/s0022-2836(83)80284-8 (1983).

3 Martinez-Garcia, E. & de Lorenzo, V. Engineering multiple genomic deletions in Gram-negative bacteria: analysis of the multi-resistant antibiotic profile of Pseudomonas putida KT2440. *Environmental microbiology* **13**, 2702-2716, doi:10.1111/j.1462-2920.2011.02538.x (2011).

4 Hernaez, M. J., Reineke, W. & Santero, E. Genetic analysis of biodegradation of tetralin by a Sphingomonas strain. *Applied and environmental microbiology* **65**, 1806-1810 (1999).

5 Santero, E. *et al.* Role of integration host factor in stimulating transcription from the sigma 54-dependent nifH promoter. *J Mol Biol* **227**, 602-620, doi:10.1016/0022-2836(92)90211-2 (1992).

6 González-Flores, Y. E., de Dios, R., Reyes-Ramírez, F. & Santero, E. The response of Sphingopyxis granuli strain TFA to the hostile anoxic condition. *Scientific reports* **9**, 6297, doi:10.1038/s41598-019-42768-9 (2019).

7 Silva-Rocha, R. *et al.* The Standard European Vector Architecture (SEVA): a coherent platform for the analysis and deployment of complex prokaryotic phenotypes. *Nucleic Acids Res* **41**, D666-675, doi:10.1093/nar/gks1119 (2013).

8 Elliott, T. & Geiduschek, E. P. Defining a bacteriophage T4 late promoter: absence of a "-35" region. *Cell* **36**, 211-219, doi:10.1016/0092-8674(84)90091-6 (1984).
